# Chemical Composition, Nutritive Value, and Toxicological Evaluation of *Bauhinia cheilantha* Seeds: A Legume from Semiarid Regions Widely Used in Folk Medicine

**DOI:** 10.1155/2013/578781

**Published:** 2013-04-17

**Authors:** Daniel Câmara Teixeira, Davi Felipe Farias, Ana Fontenele Urano Carvalho, Mariana Reis Arantes, José Tadeu Abreu Oliveira, Daniele Oliveira Bezerra Sousa, Mirella Leite Pereira, Hermogenes David Oliveira, Manoel Andrade-Neto, Ilka Maria Vasconcelos

**Affiliations:** ^1^Departamento de Bioquímica e Biologia Molecular, Universidade Federal do Ceará, 60455-970 Fortaleza, CE, Brazil; ^2^Departamento de Biologia, Universidade Federal do Ceará, 60455-970 Fortaleza, CE, Brazil; ^3^Departamento de Química Orgânica e Inorgânica, Universidade Federal do Ceará, 60455-970 Fortaleza, CE, Brazil

## Abstract

Among the *Bauhinia* species, *B. cheilantha* stands out for its seed protein content. However, there is no record of its nutritional value, being used in a nonsustainable way in the folk medicine and for large-scale extraction of timber. The aim of this study was to investigate the food potential of *B. cheilantha* seeds with emphasis on its protein quality to provide support for flora conservation and use as raw material or as prototype for the development of bioproducts with high added socioeconomic value. *B. cheilantha* seeds show high protein content (35.9%), reasonable essential amino acids profile, low levels of antinutritional compounds, and nutritional parameters comparable to those of legumes widely used such as soybean and cowpea. The heat treatment of the seeds as well as the protein extraction process (to obtain the protein concentrate) increased the acceptance of diets by about 100% when compared to that of raw *Bc* diet. These wild legume seeds can be promising alternative source of food to overcome the malnutrition problem faced by low income people adding socioeconomic value to the species.

## 1. Introduction

According to the recent FAO database, the total number of undernourished people in the world, almost 870 million, is still unacceptably high, and eradication of hunger remains a major global challenge [1]. Hence, the search for alternative food ingredients remains of utmost importance. In this context, little-known legumes may be important in terms of food security, nutrition, agricultural development, and economic growth, standing out as important factors to reduce hunger and malnutrition in developing countries. Besides their already well-described nutritional properties, such as the abundance of macro- and micronutrients, legume seeds can display nutraceutical properties which can help to prevent heart diseases, diabetes, digestive tract diseases, and obesity [2].

As in many regions of the world, the semiarid of Brazil presents a strong contrast—on one side a high biodiversity of plant species, especially the Leguminosae family with 200 described species, and on the other, a huge population with serious public health problems that could be mitigated by the sustainable use of this biodiversity [3]. In general, the legumes are underexploited by local population, being used in a nonsustainable way in the folk medicine or for other purposes. A good example in this scenario is the species *Bauhinia cheilantha* (Fabales: Caesalpiniaceae). Its aerial parts have been used in the folk medicine in many communities that live in the semiarid region of Northeastern Brazil due to their hypoglycemic properties [4]. At the same time, it is already known that among *Bauhinia *species, *B. cheilantha* stands out for its seed protein content [5], but there is no record of studies on the nutritional value of these seeds. In view of this, the present work aims to investigate the food potential of *B. cheilantha *seeds with emphasis on its protein quality to provide support for the preservation of the regional flora and use as raw material or as prototype for the development of bioproducts with high added socioeconomic value. We thus believe that by generating basic knowledge it is possible to not only promote the preservation of the regional flora but also to use it sustainably.

## 2. Materials and Methods

### 2.1. Seed Samples and Processing

Harvesting of plant material was carried out in Caatinga forest (semiarid vegetation), Northeastern Brazil. *B. cheilantha* was identified and a voucher specimen deposited at Herbarium Prisco Bezerra, Universidade Federal do Ceará (UFC), under the number EAC 43701. The seeds were separated from pods and ground into fine meal (mesh size 1.0 mm) by using a blender and a coffee mill and the meal was stored at 4°C.

### 2.2. Proximate Composition and Amino Acid Analysis

Moisture, ash, and fat contents were assayed by the Association of the Official Analytical Chemists [6], methods 945.39A, B, and D, respectively. Nitrogen was determined using the Kjeldahl method [7] and the quantity of protein was calculated as N × 6.25. The total dietary fiber was determined by Prosky-AOAC, method 985.29 [6]. The digestible carbohydrate content was determined by difference. The energy content was estimated by multiplying the percentages of crude protein, crude fat, and digestible carbohydrates by their respective modified Atwater factors, which are 17, 37, and 17 kJ/g, respectively [8].

For amino acids analysis defatted seed meals were hydrolyzed with 6 N HCl containing 10 g/L phenol at 110°C for 22 h, in sealed glass tubes under N_2_ atmosphere. HCl and phenol were removed by evaporation and the amino acid compositions were established after chromatography on a Biochrom 20 system (Pharmacia LKB Biotech AB-Uppsala, Sweden). Tryptophan was measured colorimetrically by using a standard curve with concentrations ranging from 0.05 to 0.5 *µ*g/mL of this amino acid [9]. 

### 2.3. Protein Concentrate Preparation

Seed meal was suspended (1 : 10 w/v) in 0.7 M NaCl. The suspension was maintained under continuous stirring, overnight, at 4°C, and then filtered. The filtrate was centrifuged at 12000 ×g, for 20 min, at 4°C and the clear supernatant dialyzed (cut-off 12 kDa) against distilled water. The dialyzed aqueous extract was freeze dried and called* B. cheilantha *protein concentrate. The total protein content was measured as previously described and total soluble proteins using the Bradford method [10].

### 2.4. Determination of Antinutritional and/or Toxic Protein

Haemagglutinating activity was assessed by serial twofold dilution of samples [11]. *B. cheilantha *crude extract was diluted with 0.15 M NaCl and mixed with rabbit erythrocytes (20 mg/mL suspension in 0.15 M NaCl), untreated or treated with trypsin (1 mg/mL). Trypsin inhibitor activity was determined using L-BAPNA as substrate [12]. Urease assay was carried out by minor modifications of the procedure described by Kaplan [13]. Briefly, for preparation of urease solution (50 *µ*g/mL), glycerol was added to EDTA-Phosphate buffer 0.2 M, pH 6.5, at a ratio of 1 : 4 in order to preserve this solution for about three weeks, at 4°C. In addition, to obtain the standard curve, urea was used at 0.5 M instead of 0.3 M and it was prepared in the same buffer above. Acute toxicity to mice was verified by intraperitoneal injection of samples [14]. All animal experiments were approved by the Animal Experimentation Ethics Committee of the Universidade Federal do Ceará (CEPA/UFC). 

### 2.5. Susceptibility of Antinutritional and/or Toxic Proteins to *In Vitro* Digestion

*B. cheilantha *protein concentrate (20 mg) was dissolved in 1 mL of pepsin (0.1 mg/mL in 0.1 N HCl, pH 1.8). The mixture was incubated at 37°C, for 2 h. After that, 0.5 mL was removed and an equal volume of 0.25 M Tris-HCl, pH 8.9, added to inactivate pepsin action and then submitted to haemagglutinating, urease, and trypsin inhibitory activities. The remaining 0.5 mL was added to 0.5 mL of trypsin and chymotrypsin (0.1 mg/mL of each enzyme in 0.25 M Tris-HCl, pH 8.9) and kept at 37°C, for 3 h. The mixture was tested for the same activities. The enzyme solutions were prepared to provide an enzyme/sample relation of 1 : 100 (w/w). 

### 2.6. Diets

Four diets containing *B. cheilantha *were prepared: (1) Raw *B. cheilantha* diet (Raw* Bc*); (2) Soaked* B. cheilantha *diet (Soaked* Bc*); (3) Soaked and heat treated *B. cheilantha *diet (Heated* Bc*); and (4) *B. cheilantha* protein concentrate diet (*Bc*PCd). To obtain soaked* Bc *diet, seeds were soaked for 1 h in distilled water (1 : 4, w/v) prior to grinding. The heat treatment consisted of boiling at 92°C for 60 min. This was sufficient to abolish more than 50% of urease and trypsin inhibitory activities and 100% of haemagglutinating activity. After boiling, the seeds along with the cooking water were placed in an oven at 37°C, for 24 h, followed by subsequent grinding. For comparison purposes, casein and soybean diets were offered. Diets containing *B. cheilantha* were supplemented with L*-*methionine based on the amino acid contents of seeds and protein concentrate, to meet the amino acid requirements of rats [15]. A nonprotein containing diet (NPC) was fed to allow determination of some nutritional parameters (Table 1).

### 2.7. Feeding Trials

Male Wistar rats were weaned at 21 days of age and given a commercial stock diet until their weights reached around 50 g. They were then fed the casein diet *ad libitum* for 3 days as a period of adaptation. Groups of 6 rats were placed in individual cages where they received water and diets *ad libitum* for 10 days. Rat weights, diet spillage, and refused diet were recorded daily. During the last 5 days of the experiment, feces were collected, freeze dried, weighed, and ground in a coffee grinder. At the end of the trial, the rats were killed by halothane overdose. The carcasses were dried in oven at 100°C for 72 h and ground. The nitrogen content of carcasses and feces was measured as described previously. True protein digestibility and net protein utilization (NPU) were calculated as described by Vasconcelos et al. [14]. 

### 2.8. Phytochemical Compounds

The diets were screened for phytochemical compounds such as phenols and tannins (reaction with ferric chloride), leucoanthocyanidins (heat treatment followed by alkalinization and acidification of sample), flavonoids and xanthones (reaction of magnesium granules with hydrochloric acid), steroids and triterpenes (extraction with chloroform, acetic anhydride, and sulfuric acidic), saponins (foam production after water solubilization and stirring), and alkaloids (precipitation with Hagger, Mayer and Dragendorff reagents) according to Matos [16]. These analyses were based on visual observation of color modification or precipitate formation after addition of specific reagents.

### 2.9. Statistical Analyses

Data were subjected to one-way analysis of variance (ANOVA) and the significance of differences among means was determined using the Tukey test (*P* < 0.05).

## 3. Results and Discussion

Regarding the proximate composition (Table 2),* B. cheilantha* seeds (raw, soaked, or heated) showed high protein content, which exceeds the levels found in other seeds of the same genus (16.8 to 29.3%) [17]. The protein content of *B. cheilantha* seeds is comparable or much higher than those reported for some important cultivated legume seeds such as soybean (39.5 to 44.5%) and cowpea (19.5 to 26.1%) [18, 19]. Often poor populations in the developing world depend on legume seeds, particularly beans for protein intake, mainly because the animal protein, that is considered more complete than that of plant source, is usually more expensive. In this context, the search for unconventional protein sources is very timely to analyze the feasibility of their incorporation into the diet as a way to combat the nutritional deficiencies of the poorest populations, resulting from the intake of foods that do not reach nutritional requirements [3].

The seeds showed also high content of dietary fiber, which was higher than those described for legume seeds widely consumed, such as soybean (17.4%) and black beans (22.6%) [20]. This is an interesting finding since the consumption of dietary fiber has been related to prevention of cardiovascular disease, diabetes, and digestive tract diseases, considering that it lowers the glycemix index of food as well as serum cholesterol levels [2, 21]. The detected levels of moisture, ash, lipids, and digestible carbohydrates were lower or similar to those described for legumes [3].

The levels of essential amino acids (Table 3) in the whole meal of *B. chemilantha *were lower than that of casein [22] and did not reach the requirement for rats [15]. Similar values were observed for the processed seeds. This fact was also demonstrated for soybean seeds [14]. Nevertheless, the essential amino acid levels of *B. cheilantha* protein concentrate were superior for valine, methionine + cysteine, and tryptophan and met the recommendations for 2–5 and 10–12 years old children [23], when compared to protein isolates from cowpea [24], which is one important plant protein source in Latin America as well as in many other regions of the world. This is of utmost importance considering that usually legume seeds are low in sulfur-containing amino acids (<2%) and tryptophan (<1%) [25]. 

Antinutritional/toxic activities are depicted in Table 4. The crude extract of *B. cheilantha *seeds presented trypsin inhibitory activity (31.5 gTI/Kg) similar to those of some soybean (28.5 to 62.5 gTI/Kg) and cowpea (12.0 to 30.6 gTI/Kg) genotypes [14, 19]. This activity was almost completely abolished after 90 min boiling. The haemagglutinating activity (793.6 HU/gF) determined in the seed extract was the same for both untreated and trypsin treated rabbit erythrocytes and lower than those found for soybeans (1152 to 18432 HU/gF) [18]. The heat treatment of seeds for 30 min was able to inactivate the ability to agglutinate red cells. The urease activity for the raw seeds (32.9 U/gF) is lower than that reported for soybean seeds (107.3 to 219.3 U/gF) [14]. As to acute toxicity of *B. cheilantha* extract, it was not lethal when injected intraperitoneally in mice. The low content of toxic and/or antinutritional factors adds positive attributes favoring the use of *B. cheilantha* seeds, considering that many important plant sources of protein and fiber (beans and soybeans) contain these compounds in higher quantities.

Regarding the susceptibility of the antinutritional and/or toxic proteins to *in vitro* digestion, the haemagglutinating and trypsin inhibitory activities were not detected after digestion of seed extract with pepsin. On the other hand, the urease activity was reduced from 32.9 to 10.3 U/gF. However, when the seed extract was subjected to sequential digestion with pepsin, trypsin, and chymotrypsin, there was no detected urease activity indicating that this protein only may exert its effect in the gastric mucosa of rats (data not shown). 

Considering the nutritional parameters (Table 5), the growth rate of the groups fed on raw *Bc* and soaked *Bc* was significantly similar to that observed for the NPC group. However, the animals fed on *Bc*PC and heated *Bc* showed growth rate higher than those of raw *Bc *diet. The body weights of rats fed on heated *Bc *were significantly similar to that of soybean group but lower than those fed on casein diet. The dietary intake in the experimental groups was equivalent to about 25 to 50% of that in casein group, which must have impaired animals growth. Feeding studies have showed that raw legumes did not support the growth of rats [26]. It is well known that rats reduce considerably their intake when the diet is poor in protein or has low quality proteins, which does not seem to be the case in this study. Another factor which could have interfered with food intake is the high dietary fiber content of the *B. cheilantha* seeds, which is well above 30%. In fact, it is known that dietary fiber may affect gastric emptying since it may slow gastric filling, due to its bulking and energetic dilution capacity [27]. The slow gastric emptying in turn reduces food intake. However, this does not seem to occur since dietary fiber contents of raw *Bc* and heated *Bc *were similar to each other whereas the food intake of rats on heated *Bc *was two-fold higher than those of raw *Bc. *Besides, the food intake of rats on heated *Bc *was similar to those of *Bc*PC which does not contain fiber. Similarly, the organoleptic properties of the diets can cause significant impairment of dietary intake [28]. It is likely that the poor organoleptic properties of the diets based on *B. cheilantha* meal and protein concentrate are responsible for the low dietary intake.

The NPU values (Table 5) for groups fed on raw *Bc* and soaked *Bc* diets were negative. However, the NPU values of the heated *Bc* and *Bc*PC diets were significantly similar to that of the diet based on soybean. The diet consisting of *Bc*PC showed protein digestibility value significantly similar to those of casein and soybean. The digestibility values of raw, soaked, and heated *Bc *diets were higher than those for raw cowpea (46.5–60.3%) [29]. Raw and soaked *Bc* diets had negative biological values whereas heated *Bc* (33.5%) and *Bc*PC diets (29.2%) did not differ significantly from that of soybean diet (28.5%). Concerning the parameters of protein quality (NPU, digestibility, and biological value), the results of the groups fed on heated *Bc* and *Bc*PC diets were promising, contrary to those of the treatments with raw *Bc* and soaked *Bc* diets. In general, the heat treatment of the seeds as well as the protein extraction process (to obtain the protein concentrate) increased the acceptance of diets by about 100% when compared to that of raw *Bc* diet.

As an attempt to comprehend the poor acceptance of raw *Bc* diets, phytochemical compounds were analyzed and tannins, flavonoids, xanthones, triterpenoids, saponins, and steroids were detected. In the other test diets only saponins and steroids were observed. All these compounds have been associated with poor palatability [28] and the removal of some of them must have improved the acceptance of the heated *Bc* and *Bc*PC diets. However, further studies are necessary to clarify which of these compounds must be responsible for the poor acceptance of the raw *Bc* diet by the animals.

## 4. Conclusion

These wild legume seeds can be promising alternative source of food to overcome the malnutrition problem faced by low income people, as well as to create basic sustainability elements to prevent extinction of this species. *B. cheilantha *seeds show high protein content, reasonable essential amino acids profile, low levels of antinutritional compounds, and protein quality parameters comparable to those of legumes widely used such as soybean and cowpea. Nevertheless, their organoleptic properties should be improved by technological processes, such as heating and developing protein concentrates, in order to use the full nutritional potential of these seeds.

## Figures and Tables

**Table 1:** Composition (g/Kg) of casein, soybean, NPC, and experimental diets.

Ingredients	Diets*						
	NPC	Casein	Soybean	Raw* Bc *	Soaked *Bc *	Heated *Bc *	*Bc*PCd
Maize starch	500	377.4	295.1	312.3	312.3	302.3	326.3
Glucose	150	150	150	150	150	150	150
Maize oil	150	150	132	127	127	127	150
Cellulose	100	100	100	—	—	—	100
Vitamin mix**	50	50	50	50	50	50	50
Mineral mix**	50	50	50	50	50	50	50
Casein	—	119.6	—	—	—	—	—
Soybean	—	—	242	—	—	—	—
*B. cheilantha *							
Raw	—	—	—	307	—	—	—
Soaked^*∧*^	—	—	—	—	307	—	—
Treated^†^	—	—	—	—	—	317	—
Protein concentrate	—	—	—	—	—	—	170
L*-*methionine	—	3.0	2.3	3.2	3.4	3.2	3.7
Density (KJ/g)	16.51	16.51	16.50	16.10	16.10	16.10	16.51

*Raw* Bc*: raw *B. cheilantha *diet; soaked *Bc*: *B. cheilantha *soaked diet; heated* Bc*:* B. cheilantha *heat treated diet; *Bc*PCd: *B. cheilantha *protein concentrate diet; NPC: nonprotein control.

**Vitamin mix (g/Kg): vitamin B_12_ (100%), 0.02; folic acid, 0.04; biotin (1%), 4.0; pyridoxine HCl, 0.04; thiamine HCl, 0.06; riboflavin (99%), 0.21; Ca-pantothenato (45%), 1.2; nicotinic acid, 4.0; inositol, 4.0; *p*-amino-benzoic acid, 12.0; choline chloride (50%), 24.0; maize starch, 950.43. Mineral mix (g/Kg): calcium citrate, 296.1; calcium carbonate (40%), 65.8; copper carbonate, 1.1; magnesium carbonate, 34.3; zinc carbonate, 0.48; ferric citrate, 9.1; magnesium chloride. 6H_2_O, 5.82; sodium chloride, 74.0; potassium chloride, 119.5; monobasic calcium phosphate, 108.2; dibasic potassium phosphate, 210.1; sodium fluoride, 0.48; potassium iodate 0.1; magnesium sulfate, 75.4.

^*∧*^Seeds were soaked for 1 h in distilled water (1 : 4, w/v) prior to grinding.

^†^Seeds were boiled at 92°C for 60 min.

**Table 2:** Proximate composition of *B. cheilantha *seed meal expressed as a percentage of dry matter.

Component	*B. cheilantha *seed (%)			
	Raw	Soaked	Heated	Protein concentrate
Ash	3.9 ± 0.1^a^	4.0 ± 0.1^a^	3.9 ± 0.1^a^	ND*
Crude fat	8.7 ± 0.1^a^	8.6 ± 0.2^a^	8.6 ± 0.1^a^	ND
Protein**	35.9 ± 0.9^b^	36.0 ± 0.7^b^	31.5 ± 0.2^b^	58.9 ± 1.7^a^
Total dietary fiber	45.3 ± 0.4^a^	44.4 ± 0.3^a^	43.2 ± 0.4^a^	ND
Carbohydrate^*∧*^	6.2 ± 0.1^b^	7.0 ± 0.2^b^	12.8 ± 0.1^a^	—^†^
Energy (KJ/100 g)^#^	1080.1	1049.2	1071.3	1001.3

All values are means ± standard deviation of triplicates (three analyses of the same sample).

*ND: not detected.

**N × 6.25.

^*∧*^Determined by calculating the percentile difference from all the other constituents.

^†^Carbohydrate values could not be calculated since the other constituents were not detected.

^#^The energy content was estimated by multiplying the percentages of crude protein, crude fat, and carbohydrates by their respective Atwater modified factors.

**Table 3:** Amino acid composition (g/100 g protein) of *B. cheilantha *meal seed and protein concentrate compared to amino acid requirements for children and growing rats.

		*B. cheilantha *			Child^*∧*^	
Amino acids	Casein*			Rats**		
		Raw	Protein concentrate		2–5 years	10–12 years
Essential						
Thr	6.7	3.8	4.2	4.0	3.4	2.8
Val	6.0	4.9	5.8	5.5	3.5	2.5
Ile	5.1	2.8	3.2		2.8	2.8
Leu	9.2	7.8	7.9	8.0	6.6	4.4
Lys	7.7	5.6	6.0	6.0	5.8	4.4
Phe + Tyr	10.8	8.8	8.8	9.0	6.3	2.2
Met + Cys	3.9	1.7	3.0	4.5	2.5	2.2
Trp	1.3	1.4	1.5	1.5	1.1	0.9
Nonessential						
Asx	—	9.6	9.6			
Glx	21.7	19.6	18.4			
Ser	5.8	5.8	6.4			
Gly	1.9	5.1	4.8			
Ala	2.8	4.6	4.7			
His	2.8	2.8	2.9	2.5	1.9	1.9
Arg	3.8	7.8	6.3	5.0		
Pro	5.8	7.8	6.2			

*Amino acid composition based on the AIN 93 [28].

**Coates et al. [15].

^*∧*^FAO/WHO/ONU [23].

**Table 4:** Trypsin inhibitory, lectin, urease, and toxic activities present in the crude extract from *B. cheilantha* seeds and their stability to heat treatment.

Activities	Heat treatment*			
	0 min	30 min	60 min	90 min
Trypsin inhibitory**	31.5 ± 1.9^a^	20.7 ± 0.1^b^	11.4 ± 0.4^c^	1.2 ± 0.1^d^
Lectin^*∧*^	793.6 ± 1.2	ND^†^	ND	ND
Urease^#^	32.9 ± 1.3^a^	19.7 ± 0.3^b^	7.4 ± 0.5^c^	5.2 ± 0.5^d^
Toxic***	NL^‡^	NL	NL	NL

All values are means ± standard deviation of triplicates.

*B. cheilantha seed meal was submitted to boiling at 92°C at different periods of time.

**Trypsin inhibitory activity is expressed as mg of trypsin inhibited per g of meal.

^*∧*^Lectin activity is expressed as haemagglutinating units per g of meal (UH/gF). Haemagglutinating unit is the reciprocal of the highest dilution giving a visible agglutination.

^†^ND: not detected.

^#^Urease activity is shown as units of enzyme per g of meal. The units were calculated from Sigma information that 1 g of pure enzyme contains 870.000 units.

***Toxic activity is represented as LD_50_, 50% lethal dose. One LD_50 _designates the amount of protein in g/Kg of mouse body weight producing convulsion and death of 50% of tested animals injected by intraperitoneal route.

^‡^NL: not lethal even at a dose of 1.0 g per Kg of mouse body weight.

**Table 5:** Nutritional parameters of rats fed on *B. cheilantha *seed meal and protein concentrate compared with those of rats fed on NPC, casein, and soybean diets.

Parameters	Diets*						
	NPC	Casein	Soybean	Raw* Bc *	Soaked *Bc *	Heated *Bc *	*Bc*PCd
Initial body weight^*∧*^ (g)	74.2 ± 5.6^a^	71.5 ± 4.1^a^	72.3 ± 4.2^a^	72.8 ± 4.6^a^	74.2 ± 5.7^a^	72.7 ± 5.2^a^	74.0 ± 5.9^a^
Final body weight^*∧*^ (g)	55.7 ± 4.1^d^	117.8 ± 9.8^a^	80.2 ± 6.4^b^	55.3 ± 2.2^d^	57.6 ± 4.4^d^	70.2 ± 6.5^b^	63.6 ± 4.6^c^
Daily food intake^*∧*^ (g)	5.5 ± 0.8^c^	12.3 ± 0.8^a^	7.6 ± 1.5^b^	3.2 ± 0.4^c^	4.4 ± 0.9^c^	5.9 ± 0.7^b^	6.0 ± 0.8^b^
NPU^†^ (%)	—	64.7 ± 4.3^a^	26.2 ± 3.9^b^	−40.3 ± 5.9^d^	−7.5 ± 1.8^c^	25.6 ± 1.6^b^	26.6 ± 1.7^b^
Protein digestibility^†^ (%)	—	93.8 ± 2.4^a^	92.2 ± 4.0^a^	77.2 ± 7.2^b^	70.4 ± 7.3^b^	76.5 ± 7.4^b^	91.0 ± 8.1^a^
Biological value	—	69.0 ± 4.7^a^	28.4 ± 2.8^b^	−14.7 ± 1.7^c^	−80.9 ± 7.2^d^	33.5 ± 3.2^b^	29.2 ± 2.6^b^

Values in a horizontal row with different following letters differ significantly (*P* < 0.05).

*For key to diets see Table 1.

^*∧*^Per rat.

^†^Per group of 12 rats.
